# Nasal vaccination of six squirrel monkeys (*Saimiri sciureus*): Improved immunization protocol against *Toxoplasma gondii* with a nanoparticle-born vaccine

**DOI:** 10.1016/j.ijppaw.2023.09.002

**Published:** 2023-09-07

**Authors:** François Fasquelle, Angelo Scuotto, Anaïs-Camille Vreulx, Thierry Petit, Thomas Charpentier, Didier Betbeder

**Affiliations:** a Vaxinano SAS, 84 rue du Dr Yersin, 59120, Loos, France; b Zoo de La Palmyre, 6 avenue de Royan, 17570, Les Mathes, France

## Abstract

*Toxoplasma gondii* is an intracellular protozoon found worldwide, which completes its life cycle between felids (its definitive host) and other warm-blooded animals. While the infection rarely leads to severe complications in humans, many animal species are very susceptible to this infection, for example the squirrel monkey (*Saimiri sciureus*) which is the subject of this study. Toxoplasmosis is lethal for 80% of cases in this species, and fatal outbreaks are frequently reported in zoological parks.

No efficient treatment exists, but a new vaccine prepared with maltodextrin nanoparticles containing killed *T. gondii* antigens has been tested recently in French zoos. The animals were immunized through heterologous administrations, with two nasal doses at one-month interval, followed by nasal/subcutaneous boosts thereafter. No death has been reported since the beginning of this vaccination campaign, but we felt the protocol could be simplified.

Here, an improved and less-invasive immunization protocol was evaluated on 6 *Saimiri sciureus* in the French zoo La Palmyre. It consisted of two nasal administrations at one-month interval, followed by a nasal boost at 6 months. A specific memory T-cell immunity was observed by ELISPOT after two administrations in all the animals, without humoral responses. The results suggest that 2 nasal administrations induce a protective immune response against *T. gondii* infection and might be sufficient to induce a strong Tcell memory, further improving immunity.

## Highlights

• Efficient nasal vaccine of Squirrel Monkeys against Toxoplasma gondii.
• Nasal vaccination induces a specific T-cell immunity against Toxoplasma gondii.
• Nanoparticle-born nasal vaccine against Toxoplasma gondii in zoo animals.

## Graphical abstract

**Figure:**
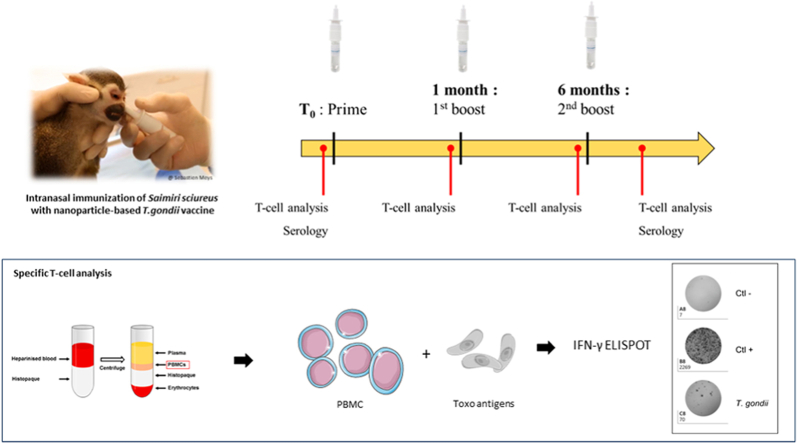

## 1 Introduction

Squirrel monkeys (*Saimiri sciureus*) are small New World monkeys belonging to the Saimiris genus and are indigenous of central and south American forests (Royo et al., 2021). As they are arboreal, mostly living in the forest canopy, their housing is increasingly endangered by the deforestation and by the fragmentation of their territory. They are therefore often found in captivity in American, European, and Asian zoos or wildlife parks for reasons of species conservation and protection, sometimes with the aim of reintroducing them back to protected areas.

Unfortunately, they appear to be sensitive to infectious diseases and particularly to *Toxoplasma gondii*, in common with many neotropical primates from south and central America (Niehaus et al., 2020). While the source of infection is most often not determined, it can occur after the ingestion of contaminated food or water and may be due to the presence of felines living in or near the zoos and shedding oocysts close to the Saimiris (Salant et al., 2009). The infection causes various symptoms such as pulmonary edema, liver and heart necrosis, and leads to death for 80% cases within a couple of days (Catão-Dias et al., 2013). This high susceptibility may come from a lack of innate and adaptive protective immunity, more commonly developed by humans, which allows the parasites to quickly reach and damage vital organs (brain, spleen, liver, lung) soon after infection (Oh et al., 2018; Nishimura et al., 2019). Many *T. gondii* infection outbreaks have been reported during the last decade in European or Asian parks, leading to important animal losses (Salant et al., 2009; Oh et al., 2018; Nishimura et al., 2019; Cedillo-Peláez et al., 2011; Pardini et al., 2015). As no efficient treatment yet exist, only preventive measures can be established in an attempt to prevent animals from ingesting contaminated feeds (Dunay et al., 2018).

Recently, a prophylactic vaccine was tested in French zoos where lethal *T. gondii* infection occurred annually (Ducournau et al., 2023). The vaccine was based on inactivated *T. gondii*, associated with lipidated maltodextrine-based nanoparticles (NPL), and was administered twice nasally at one-month intervals, followed by heterologous nasal/subcutaneous boosts 6 months, one year and two years after the beginning of the study. No death has occurred among vaccinated animals in the participating zoos since the first administration, thanks to a memory Th1 immune response (Ducournau et al., 2023).

Based on these first results, we devised and tested an improved, less-invasive immunization protocol on 6 *Saimiri sciureus* from the French zoo La Palmyre, using just 3 nasal administrations of the NPL vaccine.

## 2 Material and methods

### 2.1 Material

Roswell Park Memorial Institute medium (RPMI 1640), Dulbecco's Phosphate Buffered Saline (DPBS), Fetal calf serum (FCS), Penicillin, Streptomycin, Histopaque, and Dimethylsulfoxide (DMSO) were purchased from ThermoFisher Scientific, France. Sodium hydroxide tablets (NaOH), Glycidyltrimethylammonium chloride (GTMA), Epichlorohydrin, 2-Mercaptoethanol, phytohemagglutinin (PHA), Concanavalin A (ConA), Tween-20, Bovine serum albumin (BSA) and p-Nitrophenylphosphate (pNPP) were purchased from Sigma, France. Dipalmitoylphosphatidyl glycerol (DPPG) was purchased from Lipoid, Germany. Anti-human IgG antibody was purchased from Mabtech (Sweden).

### 2.2 Animals

Six *Saimiri sciureus* from the French zoo La Palmyre were included in this vaccination protocol, none of which were subject to the European animals’ experimentation rules (Directive, 1999/22/CE). All these animals were captive born and arrived at the zoo in June 2022. They were enclosed in an outside aviary connected to an inside exhibit enclosure. They were housed in single-species exhibit containing rocks, branches, wooden poles, ropes and a small pond. They were fed twice a day with fruits, vegetables, monkey pellets and an extra protein source (red meat, day-old chicks). Water was available *ad libitum*. No medical history was reported before their arrival nor after. They were monitored daily by zookeepers, and veterinarians had the responsibility for their well-being, health, and medical care. The *T. gondii* infection status was checked before beginning the vaccination program and at each blood sampling, by an immunochromatographic IgG-IgM test (Toxoplasma ICT IgG-IgM, LDBio, France).

### 2.3 Vaccine preparation

The vaccine was composed of inactivated *Toxoplasma gondii* tachyzoites associated to NPL.

*T.gondii* parasites were produced on Vero cells: Vero cells were first seeded in T75cm^2^ flasks with 10 mL complete RPMI 1640 (10% heat-inactivated FCS, 1% Penicillin/Streptomycin) until they reach 80% confluency. Tachyzoites were thawed at 37 °C, and 10^8^ parasites/mL were seeded into the Vero flasks. Then, Vero cells and *T. gondii* tachyzoites were subcultured in T225cm^2^ flasks. When full Vero cell lysis was reached, the supernatant was harvested. Tachyzoites were then purified from cell debris by centrifugation and washed in DPBS 3 times by centrifugation. The parasites were finally inactivated by repeated freeze/thaw cycles.

NPL were prepared under Good Manufacturing Processes (GMP), as described earlier (Paillard et al., 2010). Briefly, maltodextrin was dissolved in 2M NaOH with magnetic stirring at room temperature. Then epichlorohydrin and GTMA were added to obtain a dense, cationic gel. The gel was neutralized with acetic acid, and then crushed through a high-pressure homogenizer (LM20, Microfluidics, France). The particles thus obtained were then filtered by tangential flow ultrafiltration (AKTA flux 6, GE Healthcare, France) through a 750 kDa membrane (GE Healthcare, France), and 70% DPPG (% weight) was added by mixing in water for 2h to obtain the final NPL.

The vaccine formulation was prepared by mixing prepared NPL with killed *T. gondii* tachyzoites (3:1 wt ratio) in water, at room temperature. It was then stored at 4 °C until use. Each dose contained 50 μg of *T. gondii* antigens calculated by a BCA assay (Pierce, France), in a total volume of 50 μL.

### 2.4 Immunization

**Fig. 1:**
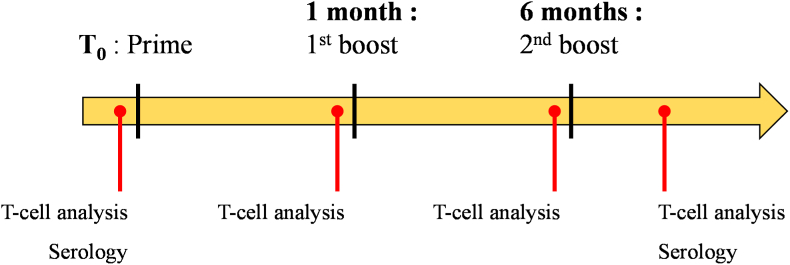
Schedule of the vaccinal protocol and of the immunological analysis performed on the 6 Saimiris.

Before each immunization, the animals were captured with a net and anesthetized by isoflurane inhalation (5% at start and 3% thereafter, 2 L/min oxygen supply). Animals were immunized intranasally with a commercial nasal spray device (VP7-50 232 NE pump, Aptar Pharma, France), containing the vaccine prepared above, three times at T_0_, 1 month and 6 months (Fig. 1).

### 2.5 Safety

After each nasal administration, the monkeys were surveyed for the appearance of eventual side effects related to the vaccination. Local side effect (sneezing, nose bleeds, runny nose or watery eyes, pruritus, alopecia, edema), anxiety and pain-related disorders (aggressiveness, lack or difficulty of movement, loss of appetite) and behavior changes (self-mutilation, unusual posture and activity, withdrawal from the group, unresponsiveness) were carefully monitored.

### 2.6 PBMC isolation

To follow the immune response induced by the vaccination, blood was sampled at varying time-point corresponding to regular animal clinical surveillance. Blood was collected from a femoral vein, and up to 2 mL of blood could be sampled at each time point.

The blood samples were collected in ethylenediaminetetraacetic acid (EDTA) tubes (BD Vacutainer K2 EDTA tube, Becton-Dickinson, France), and initially subjected to the Toxoplasma ICT IgG-IgM rapid diagnostic test to determine the animals’ infection status. Then, PBMC were isolated from these samples using a Histopaque gradient: 1 mL of blood was diluted with 1 mL of DPBS at room temperature, then slowly layered onto 2 mL of Histopaque in 15 mL conical tubes and centrifuged at 400 g for 30 min. The upper layer, containing the serum, was collected and stored at −80 °C. The mononuclear cell fraction in the opaque interface was also collected and washed twice in DPBS at 300 g for 10 min. The cells were then counted and resuspended in freezing media (RPMI with 10% FBS, 50 μM 2-Mercaptoethanol, 1% Pen/Strep and 10% DMSO) and cryopreserved at −80 °C for short term storage.

### 2.7 IFN-γ ELISpot

The T-cell immunity against *T. gondii* was assessed by ELISPOT at each time point (Fasquelle et al.). Frozen PBMC were thawed at 37 °C and washed in pre-warmed, complete RPMI 1640 (10% heat inactivated FCS, 100 IU/mL penicillin, 100 mg/mL streptomycin, 50 mM 2-mercaptoethanol) by centrifugation at 400*g* for 10 min. They were stimulated with either complete RPMI as a negative control or 10 μg/mL inactivated parasites for 48 h at 37 °C. Then, for each condition, 2 × 10^5^ cells were carefully harvested and seeded in 96-well precoated IFN-γ ELISPOT plate (Mabtech, Sweden), in 100 μL media. A positive control was added to the plate, by stimulating 2 × 10^5^ cells in 100 μL with PHA/ConA (5 μg/mL each). After 24h, the cells were discarded, the spots were revealed, and then counted on an ELISPOT reader (Astor, Mabtech, Sweden). Antigen-specific responses were expressed as spot-forming units (SFUs) per 10^6^ PBMCs after subtraction of spots in negative control wells.

### 2.8 Serology

The titration of anti-*T. gondii* serum antibodies was assessed by ELISA before the beginning of the study and after each of the 3 immunizations (Ducournau et al., 2023). A 96-well plate was coated with 100 μL per well of 10 μg/ml of inactivated *T. gondii*, in carbonate buffer at pH 9.6, overnight at 4 °C. After 3 washes with 200 μL PBS/Tween-20 0.05%, 200 μL of PBS/BSA 4% was added for 90 min at 37 °C. After 3 washes with 200 μL PBS/Tween-20 0.05%, 100 μL of sera from each animal was incubated with serial two-fold dilutions starting from 1/100 to 1/204,800 in PBS/Tween-20 0.05%, for 1 h at 37 °C. After 3 washes with 200 μL PBS/Tween-20 0.05%, 100 μL of a monkey cross-reactive anti-human IgG antibody (Mabtech, Sweden) coupled to alkaline phosphatase was added to each well, diluted at 1/1000 in PBS/Tween-20 0.05%, and left for 90 min at 37 °C. After 3 last washes with 200 μL PBS/Tween-20 0.05%, the antibody presence was revealed by the addition of a ready-to-use p-Nitrophenylphosphate (pNPP) solution. The optical density (OD) was measured at 405 nm using a microtiter plate reader (Multiskan, ThermoFisher France). The negative threshold was calculated at each serum dilution, as the mean + 2.5 x SD of three serum samples from seronegative human.

### 2.9 Statistical analysis

T-cell response over time was compared using the Kruskal-Wallis test (Friedman test for matched values could not be performed due to missing data in the final follow-up).

## 3 Results

### 3.1 Safety

A total of 6 *Saimiris sciureus* monkeys were included in the study. They were all male and aged between 1 and 4 years old at the beginning of the study (Table 1).

**Table 1:** Identification, sex and age of the 6 *S. sciureus* included in the vaccination protocol.

Monkey (ID)	Sex	Age at T0 (Y) - Date of birth
9583	M	1 - June 22, 2021
9584	M	4 - July 10, 2018
9585	M	1 - June 06, 2021
9586	M	2 - August 26, 2021
9587	M	1 - May 19, 2021
9588	M	2 - July 10, 2021

After each vaccination procedure, they were found to be healthy and in good body condition. They recovered quickly after anesthesia and no local side effect, anxiety, pain-related disorders were observed. Their behavior remained overall very stable.

### 3.2 Immune response

**Fig. 2:**
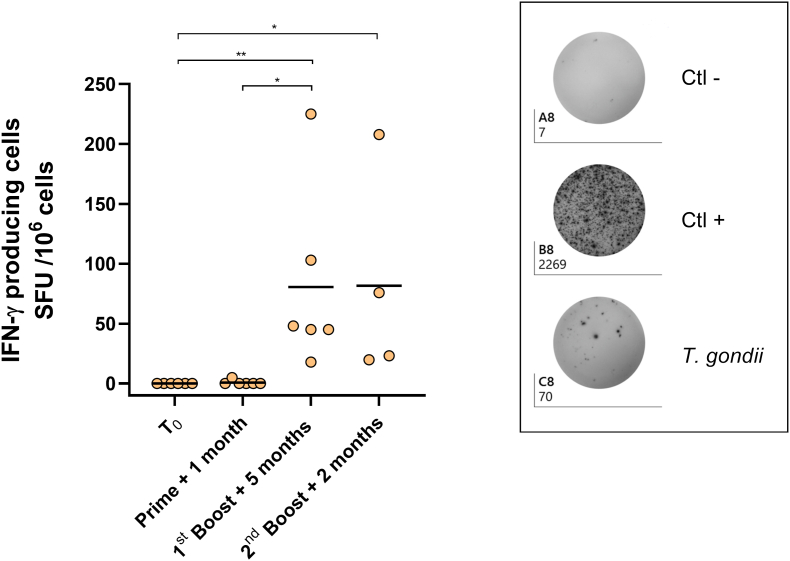
T-cell immune response analyzed by IFN-γ ELISPOT on PBMC from 6 Saimiris. The results are presented as Spot Forming Units for 10^6^ PBMC (left), before the immunization (T0), one month after the prime, 5 months after the 1st boost and 2 months after the 2nd boost. A representative picture of the ELISPOT plate after the 2nd boost is presented (right). Only 4 animals were analyzed by ELISPOT after the 2nd boost due to blood coagulation in the sampling tubes. Statistical analyses were made by Kruskal-Wallis test, * p < 0.05, ** p < 0.01.

The T-cell immune response was assessed before the first immunization and after each administration, by measuring the IFN-γ secretion of PBMC stimulated with inactivated *T. gondii* by IFN-γ ELISPOT (Fig. 2). The analysis showed no spot before the vaccination (T_0_), neither after the first administration (Prime + 1 month). However, a significantly greater number of spots was observed after the second administration (1st boost + 5 months, mean = 81 spots) confirming the establishment of a T-cell immunity. Moreover, an equivalent amount of spots was observed after the third administration (2nd boost + 2 months, mean = 82 spots) confirming the established T-cell memory.

**Fig. 3:**
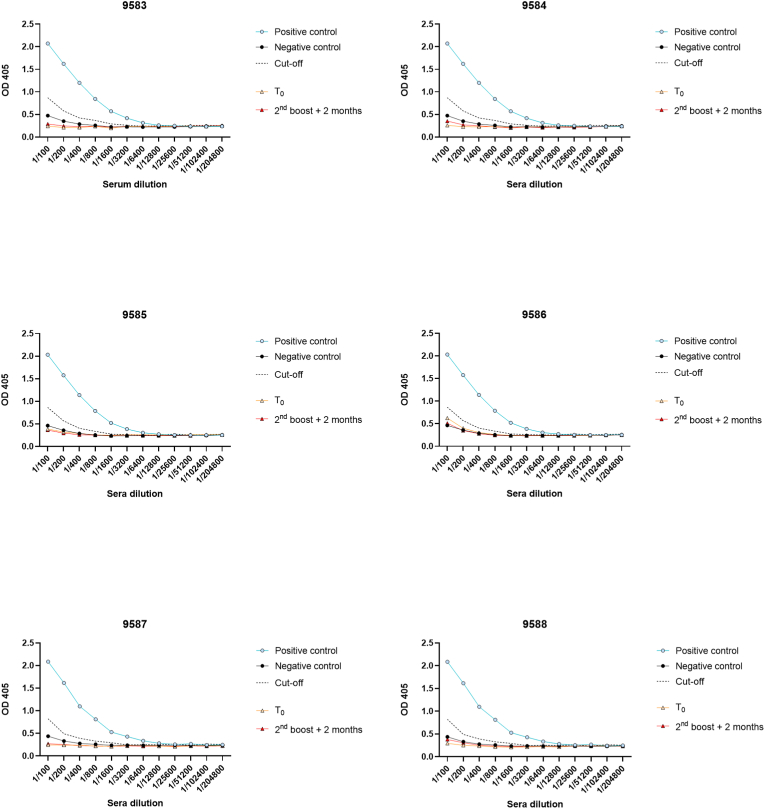
Humoral immune response analyzed by ELISA on serum for each Saimiri. The results are presented as optical density (OD) before the immunization (T0), and 2 months after the 2nd boost. Serum from one seropositive and three seronegative humans were used as positive and negative controls, respectively. Cut-off was determined at each dilution, as the mean + 2.5xSD of the negative controls.

The humoral response was evaluated as well by measuring IgG titers against *T. gondii* in the blood, before (T0) and after (2nd boost + 2 months) the immunizations (Fig. 3). The analysis revealed no increase of the optical density after the three nasal administrations, suggesting no triggering of the humoral immune response. These observations were supported by the immunochromatographic IgG-IgM tests performed at each blood sampling, which remained all negative along the study. Altogether, these results suggest the vaccine induced a pure memory T-cell response against the parasite.

## 4 Discussion

As for many animal species whose habitat is threatened, maintaining Saimiris in captivity is a means of preventing their potential extinction. It also enables scientists to study these animals in surroundings that mimic their natural environment. Unfortunately, their captivity also exposes them to pathogens they rarely encounter in the wild, and their immune system might not be able to manage these infections (Niehaus et al., 2020).

*Toxoplasma gondii* is an intracellular protozoon which occurs worldwide, perpetuating its life cycle between felids, its definitive host, and other warm-blooded animals, mainly rodents (Elsheikha et al., 2020). It is estimated that 30–50% of the human population is infected with the parasite, through the ingestion of contaminated food or water, but this rarely leads to severe complications except among immunosuppressed people or pregnant women (Dubey, 2021). As many New World primates, Saimiris are highly susceptible to this infection, and being held in captivity greatly enhances the probability they will encounter the parasite (Salant et al., 2009; Catão-Dias et al., 2013). The infection is lethal for 80% of cases and no efficient treatment exists. While daily preventive measures help to protect the animals, they can be difficult to manage, and even a slight alteration (vegetable wash, feeding with organic food …) can lead to an infection that will contaminate the whole population.

An innovative nasal vaccine against *T. gondii* has been developed for the last 10 years (Dimier-Poisson et al., 2015). The formulation is based on maltodextrin-based nanoparticles (NPL) loaded with killed *T. gondii* tachyzoites. The NPL are inert and don't trigger any immune reaction by itself, but act as a delivery system to improve the antigens mucosal delivery and consequently their immunogenicity (Bernocchi et al., 2016; Fasquelle et al., 2022). These particles were designed for the nasal route of administration, as they can cross mucus layers and enhance the mucosal residence time of antigens (Bernocchi et al., 2016; Lê et al., 2019; Fasquelle et al., 2020). Preclinical studies have been conducted on mice and sheep, which demonstrated that two nasal administrations of this formulation protected the animals against lethal infections and against the parasite vertical transmission, without any side effects (Ducournau et al., 2017, 2020).

Based on these results, the vaccination of captive Saimiris was proposed in 6 French zoos dealing with *T. gondii* infection (Ducournau et al., 2023). The vaccine was considered as an experimental prophylactic procedure which, in the absence of an alternative, could help in tackling a real health issue for these captive animals. All the decisions were taken under the zoo's veterinarians' responsibility, and this particular situation is not considered as any sort of experimentation on the animals. Forty-eight monkeys were vaccinated in this previous study, with a nasal prime/boost administration at one-month interval, followed by heterologous nasal/subcutaneous boosts at 6, 12 and 24 months. The heterologous boosts were proposed by the veterinarians to broaden the immune response. After 5 years of survey, no death related to infection by *T. gondii* was observed, while 3–26% of animals used to die each year from this infection. A Th1 immune response was induced by the vaccination, without any humoral response (Ducournau et al., 2023).

Thanks to these initial, very promising observations, more zoos decided to being part of the vaccination campaigns against toxoplasmosis. Various other animal species, including lemurs and marsupials, have been vaccinated with nasal administrations in several zoos where animals were still dying from the infection. As observed previously, deaths from *T. gondii* infection ceased in these zoos after the beginning of the vaccination campaign. However, it is laborious to follow the immune response in these species owing to the lack of recognition of IFN-γ with commercial specific antibodies. In addition, a simpler immunization protocol was suggested, aiming to reduce invasive procedures on the animals, and the immunogenicity was evaluated only on Saimiris for which cross-reactive IFN-γ antibodies are available.

In the present study, 6 *Saimiris sciureus* from La Palmyre zoo were vaccinated, with a nasal prime/boost at one-month interval, followed by a second boost at 6 months. Moreover, the T-cell memory response was assessed by an ELISPOT protocol developed in our laboratory. An increasing Th1 response was observed after two doses, with the secretion of IFN-γ by stimulated PBMC 5 months after the first boost. This suggests that two nasal administrations are sufficient to trigger adaptative cellular immunity. After a second boost, no increase in the IFN-γ secretion by PBMC was observed, suggesting no strengthening of the immunity. This second boost could nevertheless extend the duration of immunity, a hypothesis that could be confirmed with a longer-term follow up of the animals, with future blood sampling and PBMC stimulations. Concerning the humoral response triggered by the immunization, no specific IgG was detected in the serum of the animals. This suggests that the vaccine only induced a memory T-cell response, similar to what was observed in the other studies, and protective enough to prevent the animals from the infection (Ducournau et al., 2020, 2023). Obviously, this protection will only be proved when a new toxoplasmosis outbreak is reported in the participating zoos. Noteworthy, the induction of a mucosal humoral immunity, mediated by secretory IgA is also possible, despite it was never observed in the previous mice studies (Dimier-Poisson et al., 2015; Ducournau et al., 2017).

Significant weaknesses of our study include the relatively small number of animals and the fact that all the animals were young males. It would be worth analysing the immune response of female and older animals for a wider understanding of the vaccine's immunogenicity. Moreover, a long term follow up will help assessing the duration of immunity in these animals, therefore the T-cell immunity is planed to be evaluated annually, as well as the serum humoral immunity.

Finally, the vaccine was proved to be safe after nasal administration, as in all previous vaccination campaigns, with no local side effects or allergic reaction reported after immunization, and no behaviour change observed the following days.

## 5 Conclusion

This new vaccination schedule applied to *Saimiri sciureus* is less invasive and established a memory Th1 immunity against *T. gondii* after only 2 nasal administrations and for at least 6 months. A 3rd boost might be necessary to induce a strong T-cell memory. This protocol has already been applied on a large number of species with success, and no deaths from toxoplasmosis have occurred in these zoos following vaccination.

## Declaration of competing interest

The authors declare the following financial interests/personal relationships which may be considered as potential competing interests: Fasquelle reports a relationship with Vaxinano that includes: employment. Scuotto reports a relationship with Vaxinano that includes: employment. Vreulx reports a relationship with Vaxinano that includes: employment. Betbeder reports a relationship with Vaxinano that includes: employment. Betbeder has patent #WO2014041427A1 licensed to Licensee.
